# Focal Versus Combined Focal Plus Radial Extracorporeal Shockwave Therapy in Lateral Elbow Tendinopathy: A Retrospective Study

**DOI:** 10.3390/jfmk9040201

**Published:** 2024-10-22

**Authors:** Caterina Delia, Gabriele Santilli, Vincenzo Colonna, Valerio Di Stasi, Eleonora Latini, Antonello Ciccarelli, Samanta Taurone, Antonio Franchitto, Flavia Santoboni, Donatella Trischitta, Sveva Maria Nusca, Mario Vetrano, Maria Chiara Vulpiani

**Affiliations:** 1 Physical Medicine and Rehabilitation Unit, Sant’Andrea Hospital, Sapienza University of Rome, 00189 Rome, Italy; 2 Department of Movement, Human and Health Sciences, Division of Health Sciences, University of Rome “Foro Italico”, 00135 Rome, Italy

**Keywords:** extracorporeal shockwave therapy, lateral epicondylitis, rehabilitation, physiotherapy, visual analog scale, conservative treatment, PRTEE

## Abstract

**Background:** Lateral epicondylitis of the elbow, commonly known as tennis elbow, is a musculoskeletal disorder characterized by pain and degeneration of the common extensor tendon. Despite various treatments, optimal management remains debated. **Objective:** This study aimed to compare the effectiveness of focal extracorporeal shockwave therapy (F-ESWT) alone versus a combination of focal and radial pressure waves (F-ESWT+R-PW) in treating chronic lateral epicondylitis. **Methods:** This retrospective observational study included 45 patients diagnosed with chronic lateral epicondylitis divided into two groups based on the treatment received: group A (F-ESWT, n = 23) and group B (F-ESWT+R-PW, n = 22). Both groups underwent three weekly sessions of their respective treatments. Patients were also given a home exercise protocol. Primary outcomes were assessed using the Visual Analog Scale (VAS) for pain and the Patient-Rated Tennis Elbow Evaluation (PRTEE) for pain and functional impairment at baseline (T0), 4 weeks (T1), 12 weeks (T2), and 24 weeks (T3) post-treatment. Secondary outcomes included grip strength and ultrasonographic measurements of common extensor tendon (CET) thickness and vascularization. **Results:** Significant improvements in VAS and PRTEE scores were observed in both groups at all follow-up points. Group B showed greater pain reduction at T1 (VAS: 3.0 ± 1.6 vs. 4.43 ± 1.47; *p* < 0.005) and T2 (VAS: *p* < 0.030) compared to group A. Functional outcomes (PRTEE) also favored group B at T1 (*p* < 0.030) and in the pain section at T2 (*p* < 0.020). Grip strength improved similarly in both groups. CET thickness showed no significant differences at T3. Vascularization decreased significantly in both groups, with a non-significant trend favoring group B. **Conclusions:** The combined F-ESWT+R-PW therapy proved more effective than F-ESWT alone in the short- to mid-term management of chronic lateral epicondylitis, significantly enhancing pain reduction and functional outcomes. The combination of focal and radial pressure waves offers a superior therapeutic approach, leveraging the distinct mechanisms of each modality for better clinical results. Further research is needed to confirm these findings and establish long-term efficacy.

## 1. Introduction

Lateral epicondylitis of the elbow is a common musculoskeletal disorder characterized by degeneration at the origin of the extensor carpi radialis brevis muscle on the lateral epicondyle of the humerus [1]. The annual incidence in the general population is 1–3%. It frequently affects individuals aged 35 years or, with no difference in prevalence between the two sexes. The dominant side is by far the most affected [2,3]. The main risk factors, especially in sports and work environments, include overuse and repetitive movements, particularly flexion extension of the wrist (>2 h per day) and activities involving loads of more than 5 kg for more than 2 h per day, leading to progressive micro-tearing, degeneration, immature repair, and tendinosis, especially at the origin of the common extensor tendon (CET). In response to tendon healing mechanisms, nonoperative treatment for elbow tendinosis can be structured into distinct phases: protecting the tendon, improving overall arm strength, and gradually returning to activity. Each phase plays a crucial role in the holistic management of elbow tendinosis [4,5,6,7].

Histologic studies have shown that lateral epicondylitis is characterized by granulation tissue formation, fibroblastic hyperproliferation, vascular hyperplasia, unstructured collagen formation, and the absence of inflammatory cells, such as macrophages, lymphocytes, and neutrophils. This condition is better described as angiofibroblastic dysplasia [8,9,10].

The diagnosis of lateral epicondylitis is usually clinical and often confirmed by the anamnesis collection and physical examination. Patients often have lateral epicondyle pain, sometimes 5 cm distal to the insertion of the common extensor tendon of the carpus. The pain may be spontaneous, evoked by acupressure, or exacerbated by flexion extension movements of the wrist or prone supination of the forearm [11,12]. Some specific tests, such as the Cozen test, Mill test, Maudsley test, and Thomson test, are commonly used in clinical practice [12,13]. The reduction of grip strength on the affected side is considered an effective test with 83% accuracy [14]. For a differential diagnosis, we should mainly consider arthrosis, instability, cervicobrachialgia, and radial tunnel syndrome [11]. Sonography is a diagnostic imaging technique that complements clinical examination findings to assess the effectiveness of treatment and enhance the accuracy of diagnosing lateral epicondylitis (LE) [15,16,17]. It is useful to combine imaging investigations, such as ultrasonography and MRI, for confirmation and in cases of differential diagnosis [18]. Association between neovascularity and pain and function has shown that neovessels and accompanying nerves might be responsible for pain symptoms [19,20,21,22].

Regarding treatment, rest, exercise, cryotherapy, non-steroidal anti-inflammatory drugs (NSAIDs), and botulinum toxin are usually used to reduce pain and inflammation to return to daily activities [23,24,25]. The most used physical modalities are ultrasound (US), laser therapy, TECAR (Transfer of Energy Capacitive and Resistive) therapy, and extracorporeal shockwave therapy (ESWT) [26]; several therapeutic exercise regimens have been proposed for the treatment of tennis elbow. It has been found that an isokinetic eccentric exercise program was more effective compared to a non-strengthening exercise program, concluding that isokinetic exercises were superior [1].

Regarding ESWT, over the past 30 years, numerous studies have been produced in the literature on the use of this therapy for chronic LE [27,28]. Specifically, there have been studies utilizing F-ESWT alone [29] and others utilizing R-PW alone [30], comparing them in reviews and meta-analyses for chronic LE [31,32]. However, these studies have not considered the fundamental differences between these two therapies in chronic LE.

In general, there are two distinct types of ESWT: focused extracorporeal shockwave therapy (F-ESWT) and radial pressure waves (R-PWs). These differ in their physical characteristics and wave propagation patterns. The pressure wave of F-ESWT typically shows a rapid rise in pressure over a very short time. The F-ESWT beam has a concentrated shape, with the pressure converging to an adjustable focal point at a selected depth within the body tissues. In contrast, R-PW features a different linear pressure, a relatively low propagation velocity, and a longer rise time duration. The maximum pressure of R-PW is at the skin surface and diverges as it penetrates deeper. These differences can result in varying therapeutic effects between F-ESWT and R-PW [33,34,35].

There are studies that have analyzed F-ESWT individually [36,37] or R-PW individually [38], as well as studies that have combined one of these methods with laser therapy [39] or the application of a wrist-extensor splint [26], in various fields of musculoskeletal pathologies. However, no study to date has evaluated the combination of these two methods compared to each of the individual methods combined with a home exercise protocol for LE. This was the aim of our study. We chose to use F-ESWT alone versus a combination of F-ESWT and R-PW because the scientific literature widely supports the superior therapeutic capabilities of F-ESWT [40,41,42]. Nevertheless, in daily clinical practice in other anatomical districts, the combination of F-ESWT with R-PW has already been utilized [43,44,45,46].

The lack of studies comparing isolated F-ESWT therapy with the combination of F-ESWT and R-PW motivated us to conduct this research to address a gap in the shockwave treatment literature for chronic lateral epicondylitis. Therefore, the objective of this study was to compare the effectiveness of focal extracorporeal shockwave therapy (F-ESWT) alone versus the combination of F-ESWT and radial pressure waves (F-ESWT+R-PW), which are both associated with a therapeutic exercise protocol, in improving pain, function, and other clinical outcomes in patients with chronic lateral epicondylitis.

## 2. Materials and Methods

### 2.1. Study Design and Population

**Figure 1:**
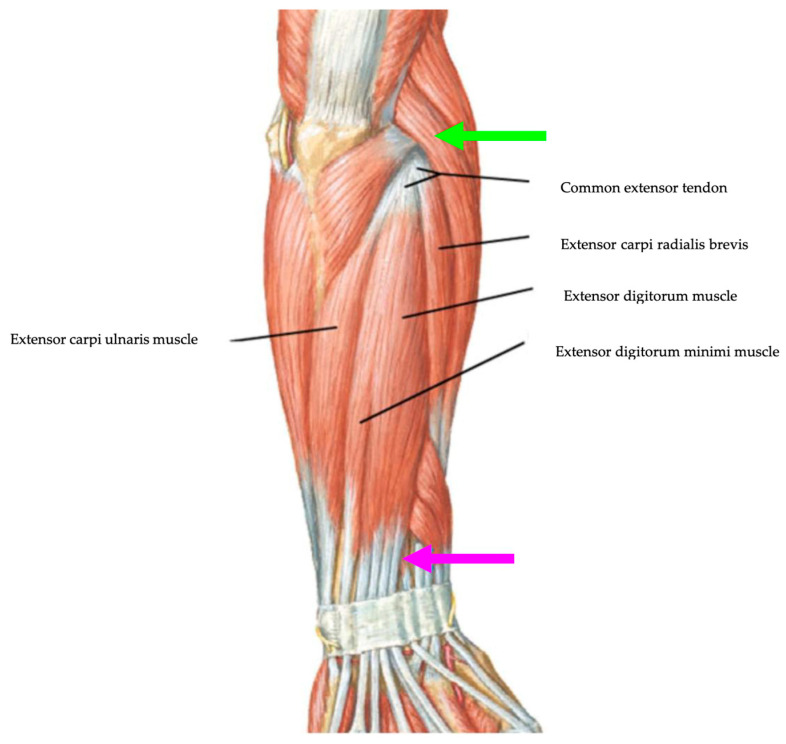
Local pain in the area of the lateral epicondyle (green arrow) and distal course of the extensor tendons (purple arrow).

**Figure 2:**
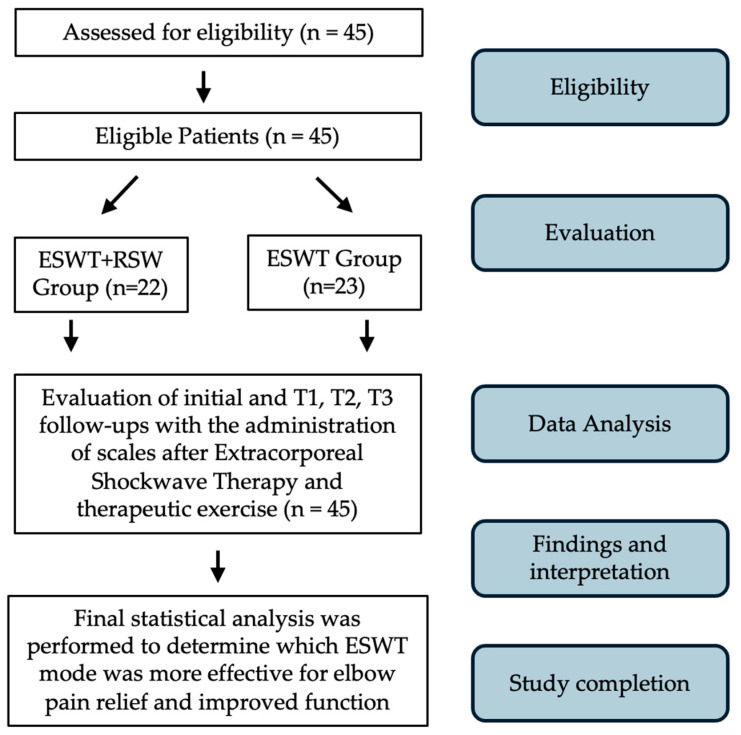
Study flow diagram.

The study protocol used was in line with the current state-of-the-art in treating LE with ESWT performed by the principal authors with the Modulith SLK system (Storz Medical, Tagerwilen, Switzerland). This observational study follows good clinical practice and the ethics of the Helsinki Declaration and was approved by La Sapienza University’s Institutional Review Board (RS 6532/2021—approval date: 15 October 2021). The informed consent of all patients has been signed, and the data have been anonymized. Data of patients treated in our institution, “AOU Sant’Andrea of Rome”, for symptomatic chronic LE between 2021 and 2024 were retrospectively collected and analyzed. Chronic lateral epicondylitis (LE) was diagnosed based on clinical symptoms and physical examinations, and the diagnosis had to be confirmed by US evaluation with MyLab™Omega Esaote (SpA, Genoa, Italy) equipped with a multifrequency linear probe of 4–15 MHz showing structural inhomogeneity and altered tendon thickness [47]. All the patients who fulfilled the following selection criteria were considered eligible: (1) lateral epicondyle pain for at least 3 months (Figure 1); (2) positive clinical tests (Cozen test and Maudsley test); (3) presence of the following ultrasound signs, abnormal echostructure, thickness at insertion, vascularization, and grade; (4) age between 18 and 75 years; (5) no previous ESWT; (6) symptoms present for last three months; and (7) both genders. Patients were excluded if they have (1) previous elbow surgery; (2) previous elbow fractures; (3) elbow instability; (4) pregnancy; (5) coagulation abnormalities; (6) rheumatologic disease; (7) physical therapy and/or CCS injections in the previous 6 weeks; (8) radial tunnel syndrome; and (9) tumor in the treatment area. All eligible patients completed a demographic and clinical questionnaire that assessed age, gender, affected side, dominant arm, and sport activity. The grip strength and scales administered at T0 and T1 after 4 weeks, at T2 after 12 weeks, and at T3 after 24 weeks from ESWT were VAS score and PRTEE score. Due to logistic reasons, the ultrasonographic measure of CET thickness and vascularization grade was collected at T0 and T3. The flow diagram of this study is shown in Figure 2. Patients were assigned to one of the groups described in the following paragraph, independently of their medical history, as both therapies have been demonstrated to be effective for chronic lateral epicondylitis in the literature [29,39] but have not been directly compared before.

### 2.2. Extracorporeal Shockwave Therapy

#### 2.2.1. Focal+Radial Extracorporeal Shockwave Therapy Group

The focal+radial group received three sessions of F-ESWT (on day 1, day 7, day 14), and they were administered by the same operator. For every single therapeutic session with F-ESWT, 2400 pulses with a range of energy flux density between 0.08 and 0.20 mJ/mm^2^ and a frequency of 5 Hz were administered. For every single therapeutic session with R-PW, 2000 pulses with a range of energy flux density between 1.4 and 3 Bar and a frequency of 20 Hz were administered. During the therapeutic procedure, patients were seated, and the affected elbow was positioned at 90° of flexion and in slight pronation. The data of patients who underwent high-energy ESWT were collected from a pre-existing dataset. All impulses were transmitted in the epicondylar area at the site of the insertion of the common extensor tendon. An ultrasound transmission gel was placed between the applicator of ESWT and the skin.

#### 2.2.2. Focal Extracorporeal Shockwave Therapy Group

The focal group received three sessions of F-ESWT (on day 1, day 7, day 14), and they were administered by the same operator. For every single therapeutic session with F-ESWT, 2400 pulses with a range of energy flux density between 0.08 and 0.20 mJ/mm^2^ and a frequency of 5 Hz were administered. During the therapeutic procedure, patients were seated, and the affected elbow was positioned at 90° of flexion and in slight pronation. The data of patients who underwent low-energy ESWT were collected from a pre-existing dataset. All impulses were transmitted in the epicondylar area at the site of the insertion of the common extensor tendon. An ultrasound transmission gel was placed between the applicator of ESWT and the skin.

### 2.3. Therapeutic Exercise Protocol

Patients in both groups followed a comprehensive rehabilitation program designed to improve flexibility and strength in the affected forearm. The stretching component aimed to maintain the physiological length of the involved muscle groups, focusing on restoring the balance between agonist and antagonist muscles. Each stretch was performed at maximum tension, without inducing pain, and held for 10–15 s. These stretches were repeated 5–10 times for 2–3 sets throughout the day. In addition to stretching, eccentric strengthening exercises were incorporated using a flexible resistance bar to enhance muscle endurance and resilience. The patients executed these exercises by holding the bar in wrist extension and, with controlled movements, applying rotational force. The wrist was progressively moved through flexion and extension, allowing the muscles to engage eccentrically. Each session consisted of 3 sets of 15 repetitions performed daily for a period of seven weeks with a 30 s rest between sets. This protocol aimed to progressively build strength and flexibility.

### 2.4. Primary Outcomes

The following outcomes were evaluated at baseline (T0) before the treatment, 4 weeks after the end of the treatment (T1), and 12 (T2) and 24 weeks after the start of the treatment (T3).

The Visual Analog Scale (VAS) comprises a 100 mm horizontal line, with “no pain” denoted at the left end (score: 0) and “pain as severe as possible” at the right end (score: 10). Patients were instructed to place a hatch mark on the line corresponding to their current pain level during their most painful movement. The VAS score was subsequently determined by measuring the distance in millimeters between the left endpoint and the patient’s mark [48].

The PRTEE included 15 items in 2 parts, a pain subscale and a function subscale. The pain subscale contained 5 items, and the function subscale had 6 items related to specific activities and 4 items related to daily activities. The responses to each item were scored on an 11-point Likert scale (0 indicating no pain or difficulty and 10 representing the worst pain or inability to perform the task). Pain scores were calculated as the mean rating across the 5 pain items. Function scores were obtained by dividing the sum of the 10 function items by 2, and the total score was calculated as the sum of the pain and function scores [49,50,51]. The validated Italian version of the PRTEE questionnaire was used in this study [50].

Grip strength was measured by a Jamar dynamometer (Jamar Hydraulic Hand Dynamometer, Sammons Preston Rolyan, Chicago, IL, USA). To measure maximum muscle strength and objectivity, the wrist was in a neutral position, the elbow flexed 90°, and the shoulder adducted and in neutral rotation with the patient seated. They were asked to “squeeze” the dynamometer as hard as possible and to hold the position for 5 s. No verbal encouragement was given during the test. Three measurements were taken at the affected side, with a minimum interval of 30 s between each measurement. Three trials were performed, and the average value in pounds (lb) of the three measurements was recorded in a database [51,52].

### 2.5. Secondary Outcomes

The thickness of the CET and bony cortex of the lateral epicondyle were assessed during sonographic imaging (while patients were seated, elbows flexed to 90°, the wrist pronated, and the arm resting on the table) [52]. All ultrasonography scannings were performed (MyLab™Omega Esaote equipped with a multifrequency linear probe 4–15 MHz) by the same sonographer with over 5 years of experience who was blinded to the treatments [53].

The degree of vascularization of the CET at the lateral epicondyle was assessed as 0, 1+, 2+, 3+, or 4+. In the absence of vessels, the tendon scored a 0. A score of 1+ was ascribed if the tendon showed 1–2 small vessels primarily around the anterior surface of the tendon. Scores of 2+, 3+, or 4+ were given if the vessel contained 2, 3, or more than 4 vessels, respectively, throughout its structure [54]. It was assessed during sonographic imaging (while patients were seated, elbows flexed to 90°, the wrist pronated, and the arm resting on the table). Ultrasonography scannings were performed (MyLab™Omega Esaote equipped with a multifrequency linear probe 4–15 MHz) by the same sonographer with over 5 years of experience who was blinded to the treatments.

### 2.6. Statistical Analysis

Power analysis was performed using G*Power (v.3.1.9.2, developed by Franz Faul and colleagues at the University of Kiel, Germany). Based on the study by Riaz S et al. [55], a desired statistical power of 90% was assumed to detect a difference of 1.5 points in the VAS pain score using a two-tailed *t*-test with Bonferroni corrections. The acceptable precision level was determined with a standard deviation (SD) of 1.5 points. A confidence level of 95% (α = 0.05) was specified, and an effect size of 0.93 was considered to determine the magnitude of practically significant differences. With these parameters, a sample size of 21 participants per group was calculated to be sufficient. Data were presented as mean and standard deviation for continuous variables and absolute frequencies and percentages for categorical variables. A non-parametric approach was considered based on the low number of patients. The Mann–Whitney U test was performed to compare the two treatment groups at 4 times (T0, T1, T2, T3). The significance of the change in each group at all follow-up times was determined by a non-parametric Wilcoxon signed-rank test. *p* < 0.05 was considered statistically significant. The statistical analysis was conducted by an external statistical consultant who was blinded to this study.

## 3. Results

Between January 2021 and August 2023, 45 eligible patients with lateral epicondylitis of the elbow were evaluated and accepted to participate. Of the 45 who wanted to participate, 23 patients were in the F-ESWT group (group A) and 22 patients were in the F-ESWT+R-PW group (group B). The study flow diagram is shown in Figure 1. The baseline demographic and clinical characteristics of patients are reported in Table 1.

No significant intergroup differences (*p* > 0.05) were found at baseline assessment. At the Wilcoxon signed-rank test, each group observed a statistically significant difference for all measures at T0 vs. T1, T0 vs. T2, T1 vs.. T2, and T0 vs. T3, except CET thickness at T0 vs. T3 and grip strength in the F-ESWT group at T1 vs. T3. A statistically significant difference was observed at T2 vs. T3 in both groups for VAS but just in group b for grip strength and the PRTEE.

The mean VAS score at T1 was significantly better in group B (3.0 ± 1.6) than in group A (4.43 ± 1.47; *p* < 0.005); also, at T2, a statistically significant difference was found *p* < 0.030) in favor of group B but not at T3.

Regarding the PRTEE, a significant difference was observed for all the sections of the PRTEE scale at T1 (tot: *p* < 0.030; function: *p* < 0.035; pain: *p* < 0.027) and at T2 only for pain (*p* < 0.020) in favor of group B.

Regarding grip strength, the mean pretreatment was 43.2 ± 14.72 for group B and 45.78 ± 15.76 for group A. Grip strength improved in both groups at all follow ups without a significant difference between them.

Regarding CET thickness, the mean pretreatment was 5.3 ± 1 for group B and 5.3 ± 0.67 for group A. Regarding vascularization grade, there was a bigger improvement in group B, but it was not statistically significant (Table 2).

## 4. Discussion

The main objective of our retrospective study was to examine, in the short and long term, the effects of combined treatment with shockwaves compared with focal shockwaves alone in patients affected by lateral epicondylitis. One of the most common upper extremity tendinopathies, epicondylitis is characterized by pain, decreased strength, and disability [1,47,56]. Among physical modalities, focused shockwaves are widely used, especially in the patient’s refractory to first-line therapies, and they have proven useful in controlling pain symptoms and long-term functional improvement in a high percentage of cases. They stimulate tissue repair through the inhibition of nociceptors, cause a “downregulation” of pro-inflammatory cytokines, and increase extracellular matrix synthesis and neoangiogenesis [26,57,58,59]. Radial pressure waves, with their decontracting action and a more superficial effect, have also been shown to be effective in decreasing pain and improving quality of life in the short to medium term (6 and 12 weeks) [23,60,61,62]. One significant issue is the heterogeneity of rehabilitation strategies, which complicates both research and clinical application [63,64,65,66]. A recent meta-analysis, including thirteen RCT studies (ten studies on F-ESWT, three studies on RSW), showed an improved grip strength and significant pain reduction in patients treated with ESWT without comparing focused or radial, but both were compared with the control group [31]. To date, no studies have been conducted on combination therapies (F-ESWT and R-PW) with shockwaves to treat chronic lateral epicondylitis.

Considering the clinical efficacy of both treatments, it has been suggested that combining the two types of shockwaves may be an effective treatment option [62]. In the literature, there are two studies on combined shockwaves in the management of Achilles tendinopathy. For Achilles tendinopathy, the combined treatment as a therapy that provides more predictable functional gains compared to radial pressure waves alone was proposed [67], and in another study, it was affirmed that the combined treatment is a safe and better method compared to the only use of F-ESWT or R-PW [68].

Regarding therapeutic exercise, some studies have shown that eccentric exercise provided improvement in pain, strength, and function. Better outcomes have been seen in programs lasting more than 6 weeks, but there is no consensus on intensity, duration, and frequency [68,69,70,71]. Combination with other therapies, such as shockwaves, facilitates the achievement of therapeutic goals. Current evidence suggests that the combination of therapeutic exercise and thermal therapy with ESWT for lateral elbow epicondylitis is an effective therapy [71,72]. In our study, patients in both groups were prescribed a detailed home exercise regimen that included stretching and eccentric strengthening exercises. At one-month follow up (T1), our patients performed the exercise protocol at a rate of 88%, at three months follow up (T2) at a rate of 85%, and at six months follow up (T3) at a rate of 78%; the adherence rates were quite high, which could explain the significant improvements in VAS and PRTEE scores at these time points, especially in group B.

Grip strength improved in both groups at all follow ups, without a significant difference between them. Although there was no significant difference in grip strength between groups, the additional mechanical stimulation provided by the combined shockwave therapy and the exercises likely enhanced the healing environment of the tendons, potentially accelerating recovery.

In our study, we did not show a significant change in CET thickness at T3 (6 months follow up). A significant decrease in CET thickness after 6 weeks by the end of the treatment in only the ESWT group was found in [52], but another study did not show a significant change in CET thickness at the 6-month follow up in the ESWT, local steroid injection, and classic physiotherapy groups compared to the baseline measurements [3]; therefore, this aspect will need to be clarified in future studies with larger sample sizes or a longer follow ups. The vascularization degree decreased significantly in both groups at T3, and no significant difference between the two groups was found. Although ultrasonographic measurements showed a non-significant trend favoring group B in reducing vascularization, it is possible that the mechanical loading from eccentric exercises, combined with shockwave therapy, helped modulate neovascularization. This supports the hypothesis that therapeutic exercises combined with shockwave therapy may enhance clinical outcomes when paired with effective shockwave treatments. However, given the lack of significant differences in tendon thickness and vascularization at T3, it is crucial to investigate the long-term effects of exercise adherence and the intensity of the prescribed regimen. In a study concerning the evaluation of vascularization in tendinopathic CET, it was noted that there was an increase in vascularization during the acute period when tendinopathy was more painful in patients [73]. For this reason, given our results showing a decrease in vascularization following 6 months after therapy with F-ESWT alone or combined with R-PW, we can state that both focused ESWT and radial pressure waves in the treatment of chronic lateral epicondylitis led to a reduction in the vascularization of the common extensor tendon, which is also linked to a decrease in VAS and the PRTEE in the short, mid, and long terms.

We found a significant difference in VAS scores between the groups in the short- and mid-term (T1 and T2), favoring the combined F-ESWT+R-PW group and allowing us to state that in the short and mid-term, the combined therapy is more effective in achieving a better outcome in terms of pain reduction of chronic lateral epicondylitis. Focal shockwaves primarily target deep tissue and stimulate tendon healing, while radial waves cover a broader area, improving circulation and reducing muscle tension around the tendon. This dual approach likely creates a synergistic effect, addressing both tendon degeneration and the surrounding soft tissue, which helps reduce pain more effectively. Additionally, the broader neurostimulation provided by radial waves may accelerate pain relief by desensitizing pain pathways. These combined mechanisms could explain the faster and more significant improvements in VAS scores observed in the combined therapy group.

At T1, all the sections of the PRTEE scale showed a significant difference in favor of the combined group, reaching the secondary outcome and allowing us to state that in the short term, the combined therapy is more effective in achieving a better outcome in terms of symptom severity and functional impact of chronic lateral epicondylitis.

Our study has shown that a combined treatment of shockwaves (F-ESWT+R-PW) offers more advantages compared to F-ESWT alone in the short–middle term. The limitations of this study may be represented by the small sample size, the absence of a follow up of >6 months after the treatment, and the lack of MRI control before and after treatment [74]. Future studies should include a broader age range to assess the variability in rehabilitation outcomes across the lifespan, which could provide insights for more personalized and age-specific rehabilitation interventions and should have larger cohorts to also allow imaging assessments using new methods, such as machine learning [75,76,77,78,79]. There are no studies in the literature about the combined treatment with ESWT for chronic lateral epicondylitis. For these reasons, additional investigations are necessary to confirm our results.

## 5. Conclusions

Our retrospective study has demonstrated that combined extracorporeal shockwave therapy (F-ESWT+R-PW) is more effective than focal shockwave therapy (F-ESWT) alone in the short- to mid-term management of chronic lateral epicondylitis. The combined treatment significantly improved pain reduction and functional outcomes, as evidenced by the VAS and PRTEE scores.

Both treatments showed a significant decrease in vascularization of the common extensor tendon at the six-month follow up, indicating a reduction in symptom severity and functional impairment. However, no significant differences in CET thickness were observed between the two groups at T3. Future studies are needed to better assess the effects on CET, especially in the long term. Our findings suggest that the combination of focused and radial pressure waves offers a more comprehensive therapeutic approach, capitalizing on the distinct mechanisms of action of each modality. This combined therapy leads to better clinical outcomes compared to focused ESWT alone. The integration of radial pressure waves with focused shockwaves represents a promising enhancement in the treatment strategy for chronic lateral epicondylitis, improving both pain management and functional recovery. Future studies using cellular morphology, microscopic, and genetic approaches are needed to better evaluate and explain the reasons for this difference in effectiveness, especially in the long term, and to establish the long-term efficacy and safety of this combined therapeutic approach.

## Figures and Tables

**Table 1:** Demographic characteristics of both groups.

Characteristics	ESWT Group	ESWT+RSW Group	*p*-Value
**Patients**, No.	23	22	
**Age**, mean + SD, years	50 ± 9.2	49 ± 8.8	0.891
**Sex**, No. (%) Male Female	15 (65.2) 8 (34.8)	9 (41) 13 (59)	
**Side**, No.(%) Right Left	17 (74) 6 (26)	16 (73) 6(27)	0.928
**Patients affected in the dominant arm**, No. (%)	18 (81)	18 (78)	0.945
**Sport activity**, No. (%) None Amateur Agonism	7 (30.4) 16 (69.6) 0 (0)	12 (54.5) 9 (41) 1 (4.5)	0.119
**Previous treatments**, No. (%)	17 (74)	12 (54)	0.175

**Table 2:** Comparison between baseline and post-treatments of the groups.

Outcome	Group	T0 Baseline Mean (SD)	T1 Mean (SD)	T2 Mean (SD)	T3 Mean (SD)
	**ESWT+RSW**	7.0 ± 1.22	3 ± 1.6	1.90 ± 1.44	1.24 ± 1.54
**VAS**	**ESWT**	6.43 ± 1.3	4.43 ± 1.47	2.91 ± 1.70	2.04 ± 2.14
***p*-value**		0.212	0.005 *	0.030 *	0.247
**Grip strength (Ib)**	**ESWT+RSW**	43.2 ± 14.72	47 ± 15.55	50.06 ± 15.15	52.54 ± 13.34
	**ESWT**	45.78 ± 15.76	52 ± 12.90	56.30 ± 11.25	56.40 ± 15.63
***p*-value**		0.633	0.617	0.323	0.385
	**ESWT+RSW**	51.45 ± 17.41	22.93 ± 16.27	14.45 ± 14.88	8.66 ± 9.40
**PRTEE tot** ***p*-value**	**ESWT**	51.2 ± 22.52	32.73 ± 16.73	21.85 ± 16.54	20.15 ± 19.25
		0.973	0.032 *	0.069	0.074
	**ESWT+RSW**	23.31 ± 8.55	9.55 ± 7.40	6.14 ± 6.70	3.78 ± 4.02
**PRTEE function**	**ESWT**	23.9 ± 12.0	14 ± 8.30	9.22 ± 8.03	7.58 ± 7.37
***p*-value**		0.919	0.035 *	0.172	0.118
	**ESWT+RSW**	27.67 ± 9.75	13.33 ± 9.53	8.31 ± 8.74	5.05 ± 5.83
**PRTEE pain *p*-value**	**ESWT**	27.35 ± 11.4	19.26 ± 9.30	14.17 ± 9.98	11.26 ± 10.50
		0.946	0.027 *	0.020 *	0.052
	**ESWT+RSW**	5.3 ± 1			5.2 ± 0.93
**CET thickness (mm)**	**ESWT**	5.3 ± 0.67			5.3 ± 0.88
***p*-value** **vascularization** **grade**		0.312			0.269
	**ESWT+RSW**	1.3 ± 1.3			0.4 ± 1
	**ESWT**	0.7 ± 1.2			0.3 ± 1.2
***p*-value**		0.078			0.854

Abbreviations: VAS, Visual Analogic Scale; SD, standard deviation; PRTEE, Patient-Rated Tennis Elbow Evaluation Scale; CET, common extensor tendon; T0, baseline; T1, 4 weeks after treatment ends; T2, 12 weeks after treatment starts; T3, 24 weeks after treatment starts; * statistical significance, *p* < 0.05.

## Data Availability

The datasets used and data analyzed during the current study will be made available upon reasonable request to the corresponding author (G.S.).
